# Prediction of gene expression with cis-SNPs using mixed models and regularization methods

**DOI:** 10.1186/s12864-017-3759-6

**Published:** 2017-05-11

**Authors:** Ping Zeng, Xiang Zhou, Shuiping Huang

**Affiliations:** 1 Department of Epidemiology and Biostatistics, Xuzhou Medical University, 209 Tongshan Rd, Xuzhou, Jiangsu 221004 China; 2 Department of Biostatistics, University of Michigan, 1415 Washington Heights, Ann Arbor, MI 48104 USA

**Keywords:** Gene expression, Cis-SNPs, Prediction model, Linear mixed model, Lasso, Elastic net, Bayesian sparse linear mixed model

## Abstract

**Background:**

It has been shown that gene expression in human tissues is heritable, thus predicting gene expression using only SNPs becomes possible. The prediction of gene expression can offer important implications on the genetic architecture of individual functional associated SNPs and further interpretations of the molecular basis underlying human diseases.

**Methods:**

We compared three types of methods for predicting gene expression using only cis-SNPs, including the polygenic model, i.e. linear mixed model (LMM), two sparse models, i.e. Lasso and elastic net (ENET), and the hybrid of LMM and sparse model, i.e. Bayesian sparse linear mixed model (BSLMM). The three kinds of prediction methods have very different assumptions of underlying genetic architectures. These methods were evaluated using simulations under various scenarios, and were applied to the Geuvadis gene expression data.

**Results:**

The simulations showed that these four prediction methods (i.e. Lasso, ENET, LMM and BSLMM) behaved best when their respective modeling assumptions were satisfied, but BSLMM had a robust performance across a range of scenarios. According to *R* ^2^ of these models in the Geuvadis data, the four methods performed quite similarly. We did not observe any clustering or enrichment of predictive genes (defined as genes with *R* ^2^ ≥ 0.05) across the chromosomes, and also did not see there was any clear relationship between the proportion of the predictive genes and the proportion of genes in each chromosome. However, an interesting finding in the Geuvadis data was that highly predictive genes (e.g. *R* ^2^ ≥ 0.30) may have sparse genetic architectures since Lasso, ENET and BSLMM outperformed LMM for these genes; and this observation was validated in another gene expression data. We further showed that the predictive genes were enriched in approximately independent LD blocks.

**Conclusions:**

Gene expression can be predicted with only cis-SNPs using well-developed prediction models and these predictive genes were enriched in some approximately independent LD blocks. The prediction of gene expression can shed some light on the functional interpretation for identified SNPs in GWASs.

## Background

In the last decade tens of thousands of SNPs have been identified by genome wide association studies (GWASs) for many complex human diseases and traits [1–3], such as type I and II diabetes [4–7], lung cancer [8–11], Crohn’s disease [12, 13], rheumatoid arthritis [13–18], blood pressure and hypertension [19–21], prostate cancer [22–26], height [27, 28], schizophrenia and bipolar disorder [29], and many others. These successes offer unprecedented insights into the genetic architectures of human diseases and traits, and may lead to clinically promising preventions and treatments for diseases in the future [30, 31]. However, the majority of identified SNPs in GWASs are located outside the protein-coding regions and their causal genetic mechanisms remain largely unknown. One way to explain this is that the identified SNPs are associated with molecular-level traits, such as methylation levels and gene expression levels, which are thought to mediate the effects of SNPs on many complex traits and diseases, and hold the key to understand the genetic basis of disease susceptibility and phenotypic variation. Recently, molecular QTL mapping have gained increasing attention [32–37], and have revealed that many cis-regulatory SNPs are not only related to diseases but also have influences on molecular phenotypes [37–39], e.g. gene expression levels which are quantitative molecular traits and can be influenced by cis-regulatory variants.

It has been found that gene expression in human tissues is heritable [38, 40, 41], meaning that predicting gene expression using only genetic variants is feasible. Gene expression levels can be effectively incorporated into models in a direct manner or in a mediated manner [42, 43], leading to a higher power for association and prediction. Additionally, accurate prediction of gene expression is a crucial step for transcriptome-wide association studies [34, 44] which attempt to construct a more biologically meaningful relationship between genes and diseases. Therefore, in addition to being significant interest in its own right for examining the relationship between SNPs and gene expression levels, knowledge of genetic variations in gene expression is also useful and important for association studies as well as phenotypic prediction [45]; integrative analysis of these information can result in a more accurate and powerful risk prediction and makes an advance towards to the precision medicine and personalized treatment of diseases. Most recently, it has been shown that, based on effective predicted values of gene expression, more powerful and interpretable gene-set tests in GWASs can be constructed [34]. Therefore, investigation of gene expression measurements can offer important implications on the genetic architecture of individual functional associated SNPs and provide further interpretations of the molecular basis underlying human diseases [32, 35, 37, 38].

Predicting complex phenotypes using genome-wide SNPs simultaneously has been increasingly used for human diseases and traits as well as animal and plant breeding [46–51], whereas predicting gene expression using SNPs is currently little studied. Based on regularized models it has recently been demonstrated [52, 53] that for some genes their expression measurements can be successfully predicted using only cis-SNPs, which are defined as SNPs located nearby a gene. In this paper we explore to predict gene expression with only cis-SNPs by borrowing two risk prediction models that are well studied and widely used in GWASs, i.e. linear mixed model (LMM) [46, 54–57] and Bayesian sparse linear mixed model (BSLMM) [58]. We evaluate the prediction performance of LMM and BSLMM with gene expression levels as phenotypes and compare them with the regularized models (i.e. Lasso and elastic net) previously employed in [34, 52, 53]. We use the Geuvadis gene expression data as an illustrative example.

## Methods

### Overview of Lasso, Elastic Net, LIMM and BSLMM

We first give a brief overview of the four methods (i.e. Lasso, elastic net, LMM and BSLMM) for predicting gene expression using only cis-SNPs. These methods are widely employed in phenotypic prediction of human complex traits and genomic selection in plant and animal breeding [51, 54, 56–68]. Compared with other methods, such as polygenic scores [29] and stepwise models, the four methods mentioned above have many advantages, e.g. they are numerically stable [69], can analyze all variants jointly while avoiding model over-fitting, and incorporate the information of linkage disequilibrium (LD); thus they have the potential to improving prediction accuracy.

Let **y** be an *n*-vector of gene expression measured on *n* individuals and assume it is centered; **X** is an *n* by *p* matrix of genotypes for *p* cis-SNPs. Lasso [70] and elastic net (ENET) [71] are both popular regularization regressions, which select important cis-SNPs and estimate their effects simultaneously by imposing a penalty [34, 52, 63] on the cis-SNPs effect sizes. Specifically, Lasso and ENET fit the following linear model

1 $$ \begin{array}{c} Q\left(\boldsymbol{\beta} \right)=\frac{1}{n}{\left(\mathbf{y}-\mathbf{X}\boldsymbol{\beta } \right)}^{\prime}\left(\mathbf{y}-\mathbf{X}\boldsymbol{\beta } \right)+{\displaystyle \sum_{j=1}^p{P}_{\uplambda}\left(\left|{\beta}_j\right|\right)},\\ {}\mathrm{Lasso}:\kern0.5em {P}_{\uplambda}=\uplambda \left|\beta \right|,\\ {}\mathrm{ENET}:\kern0.5em {P}_{\uplambda}=\uplambda \left(\alpha \left|\beta \right|+\left(1-\alpha \right){\beta}^2\right),\end{array} $$

where *P*_λ_ is the penalty function, λ is the turning parameter controlling the extent of shrinkage, and *α* provides a mix between ridge regression and Lasso [70–73]. We ignore the intercept term in the model due to the fact that **y** is centered. The coordinate descent algorithm [74, 75] is employed to efficiently fit Lasso and ENET, and λ is typically selected via *k*-fold cross validation [72]. Due to *P*_λ_, small effects will be exactly estimated to be zero with reasonably selected turning parameter. Therefore, in this sense Lasso and ENET are sparse models. In contrast, LMM [46, 54–57] assumes every cis-SNP influences the gene expression measurements, with the effects sizes following a normal distribution

2 $$ \begin{array}{c}\mathbf{y}=\mathbf{X}\boldsymbol{\beta } +\boldsymbol{\varepsilon}, \kern0.5em \boldsymbol{\varepsilon} \sim N\left(0,{\sigma}_e^2{\mathbf{I}}_n\right),\\ {}{\beta}_j\sim N\left(0,{\sigma}_b^2{\sigma}_e^2/ p\right).\end{array} $$

Again we ignore the intercept term here. In model (2) *σ*_*e*_^2^ is the residual variance, **I**_*n*_ is an *n*-dimensional identity matrix, and *σ*_*b*_^2^ is the genetic variance scaled by *σ*_*e*_^2^. Note that the narrow-sense heritability *h* ^2^ can be defined as *σ*_*b*_^2^/(*σ*_*b*_^2^ + 1) [55]. Because of assuming all variants have nonzero impacts on gene expression, LMM is thus a polygenic model [58, 76, 77]. We adopt the restricted maximum likelihood method to fit LMM using the efficient GEMMA algorithm [58, 78]. In GWASs, a few variants have displayed much larger effects than other SNPs. For example, the markers in major histocompatibility complex (MHC) region [79] in chromosome 6 show strong effects on some autoimmune diseases [13], e.g. type I diabetes, Crohn’s disease and rheumatoid arthritis. To consider this, BSLMM [58] extends LMM by additionally incorporating SNPs with stronger effect sizes into the model. That is, BSLMM models the gene expression using

3 $$ \begin{array}{c}\mathbf{y}=\mathbf{X}\tilde{\boldsymbol{\beta}}+\mathbf{u}+\boldsymbol{\varepsilon}, \kern0.5em \boldsymbol{\varepsilon} \sim N\left(0,{\sigma}_e^2{\mathbf{I}}_n\right),\\ {}\mathbf{u}\sim N\left(0,\mathbf{K}{\sigma}_b^2{\sigma}_e^2\right),\\ {}\tilde{\beta}\sim \pi N\left(0,{\sigma}_a^2{\sigma}_e^2\right)+\left(1-\pi \right){\delta}_0,\end{array} $$

where **K** is the relatedness matrix, $\tilde{\beta}$ is the large SNP effect size, *π* is probability that SNPs have large effect sizes, **u** can be viewed as the collection of small effects sizes, *σ*_*a*_^2^ is the corresponding variance, and *δ*_0_ is a point mass at zero. BSLMM is essentially a hybrid of LMM and sparse model via a spike and slab prior on affect sizes rather than imposing a penalty. In the special case of **K** = **XX** ^*T*^/*p*, we can decompose the small effects sizes as **u** = **Xβ** with ${\beta}_j\sim N\left(0,{\sigma}_b^2{\sigma}_e^2/ p\right).$ Based on re-parameterization [58], BSLMM is efficiently fit using Monte Carlo Markov Chain (MCMC) sampling. As BSLMM includes both LMM and sparse model as special cases, thus it is expected to enjoy both the advantages of LMM and sparse model.

### Simulations

We compared the performance of Lasso, ENET, LMM and BSLMM using simulations. To make our simulations much close to the real data, we used genotypes of gene *TPRG1L* from the Geuvadis program [80]. Briefly, there were a total of 465 individuals and 5,818 SNPs (minor allele frequency, or MAF, ≥0.05) in *TPRG1L*. We simulated gene expression **y** under three scenarios: (I) In addition to including all 5,818 SNPs into model as causal markers (the polygenic part), we also selected either 5 or 15 SNPs randomly with relatively large effect sizes (the sparse part). We simulated the effect sizes of the two parts from standard normal distributions and scaled the effects in each part separately so that the proportion of variance of gene expression explained (PVE) [58] by the two parts was 0.60 and 0.40, respectively. This scenario corresponded to the BSLMM modeling assumption. (II) We only modeled the polygenic part, i.e. all the SNPs were contained in the model with effect sizes following a standard normal distribution, corresponding to the LMM modeling assumption. (III) We only modeled the sparse part, i.e. again only either 5 or 15 SNPs with relatively large effect sizes were contained in the model, corresponding to the sparse modeling assumption in Lasso and ENET. In all the three scenarios the total PVE was set to 0.10, 0.30 or 0.50. In each scenario, we performed 20 simulation replicates. In each replicate, we randomly split the simulated data into a training data with 80% individuals and a test data with the rest 20% individuals. We then fit Lasso, ENET, LMM and BSLMM on the training data and assessed their performance in the test data. The performance was measured by the squared correlation coefficient (*R* ^2^) between the predicted values and the observed values in the test data. Both Lasso and ENET were implemented via the R package glmnet (version 2.0–5) [75], the penalty parameters in Lasso and ENET were selected using 100-fold cross validation. Additionally, we set *α* = 0.5 in ENET as done in [34]. LMM and BSLMM were implemented via the GEMMA software (version 0.94) [58, 78]. For BSLMM we set both burn-in and MCMC sampling sizes to 10,000.

### Application to the Geuvadis data

The Geuvadis project [80] performed mRNA and small RNA sequencing on 465 Epstein-Barr-virus-transformed lymphoblastoid cell line samples from five populations. The genotype data was from the 1000 Genomes project [81]. Since the original gene expression measurements were read counts, the PEER normalization [82–84] was employed to remove technical variations and batch effects. We quantile-normalized every gene expression to a standard normal distribution separately in the five populations and then quantile-normalized together. According to GENCODE (release 12) [85], in the Geuvadis data we selected 15,810 genes that were expressed in at least half of the individuals. For each gene we only included common cis-SNPs (MAF ≥ 0.05) that were located within the gene or in the 1 Mb upstream and downstream regions near that gene, resulting in an average of about 580 SNPs per gene. Note that here only cis-SNPs are used due to the following reasons. First, it has been found that most expression quantitative trait loci (eQTL) are near the regulated gene and only a few eQTLs are trans-acting [33, 86]. Second, the effects of trans-SNPs are usually too weak to be detected with a reasonably high power [87]. Third, incorporating trans-SNPs into the model (e.g. using a two-variance-component model [88]) may improve the predictive accuracy, but with limited sample sizes the model fitting will become difficult and may lead to numerical issues. We randomly split each gene expression in the Geuvadis data into a training data with 80% individuals and a test data with the rest 20% individuals. We then fit Lasso, ENET, LMM and BSLMM on the training data and assessed their performance in the test data. Lasso and ENET were conducted using the R package glmnet (version 2.0–5) [75]. The penalty parameters of Lasso and ENET were selected via 100-fold cross validation. LMM and BSLMM were implemented via the GEMMA software (version 0.94) [58, 78]. For BSLMM we set burn-in to 2,000 and MCMC sampling size to 10,000.

## Results

**Fig. 1:**
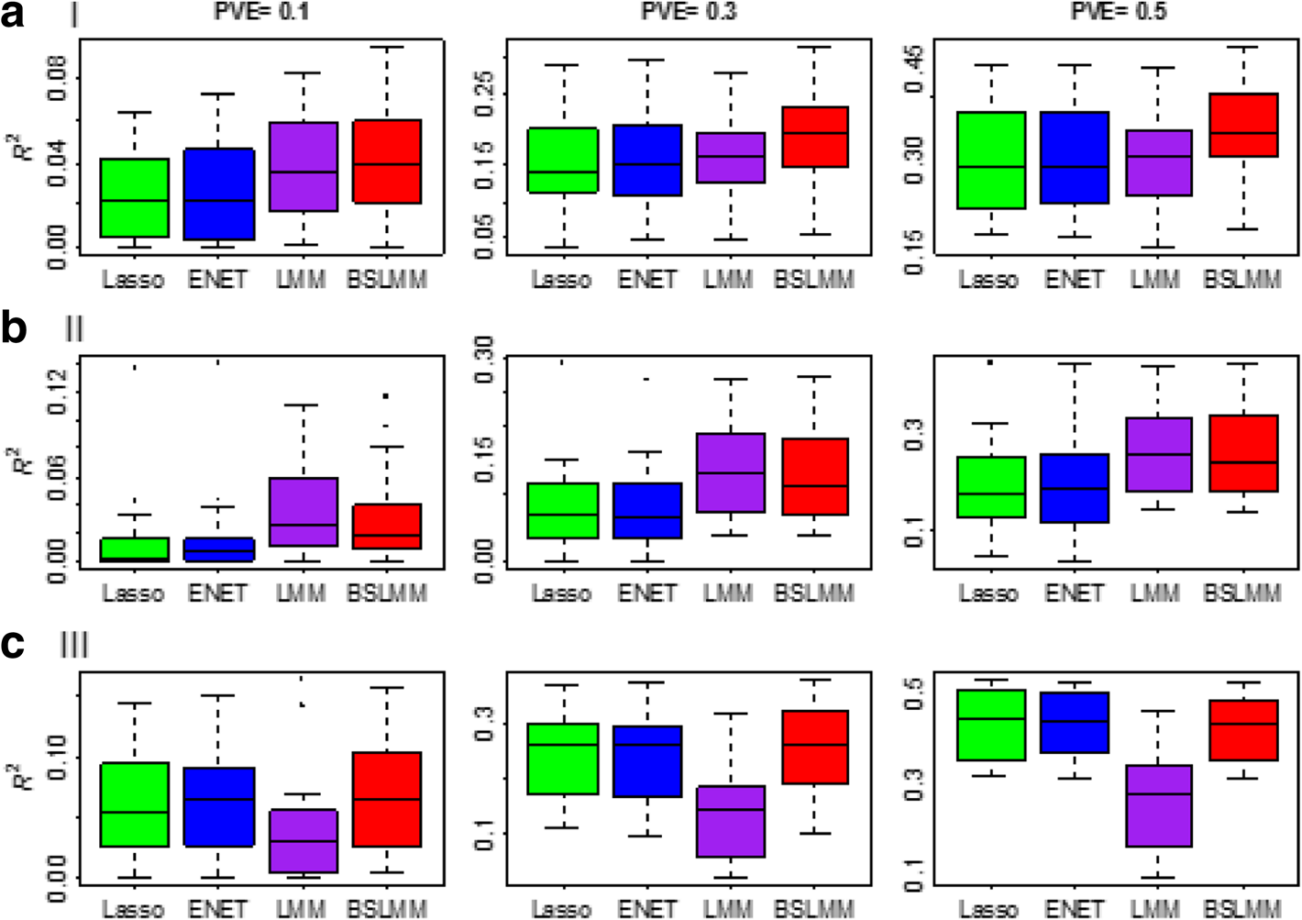
Comparison of the four methods (i.e. Lasso, ENET, LMM and BSLMM) for predicting gene expression in scenarios I-III. **a** The results of scenario I where the BSLMM modeling assumption is satisfied and 15 causal SNPs are included in the sparse part. **b** The results of scenario II where the LMM modeling assumption is satisfied. **c** The results of scenario III where the sparse modeling assumption is satisfied and there are only 15 causal SNPs and the rest are all neutral. The performance is measured by *R* ^2^. In each panel from left to right it corresponds to PVE = 0.1, 0.3 or 0.5 respectively

The simulations show that these four prediction methods behave best when their individual modeling assumptions are satisfied. (The patterns are very similar for the two cases that there were 5 or 15 causal SNPs with relatively large effect sizes in scenarios I and III, so only results for 15 are displayed) For example, in scenario I where the BSLMM modeling assumptions were satisfied (Fig. 1a), BSLMM outperforms the other methods, whereas in scenarios II and III, as expected, the best methods are LMM and Lasso (or ENET), respectively. When the underlying model assumptions are not satisfied, LMM and Lasso (or ENET) are subject to reductions of prediction accuracy; for example, LMM in scenario II (Fig. 1b) and Lasso (or ENET) in scenario I or II (Fig. 1b and c). In contrast, BSLMM is very robust across various scenarios and has a compatible performance with the best method in scenarios II and III. For instance, BSLMM is only slightly worse than LMM in scenario II (Fig. 1b) where only polygenetic effect sizes were simulated, and behaves similarly to Lasso (or ENET) in scenario III (Fig. 1c) where only sparse effect sizes were included.

To compare the speed of these methods, we selected seven genes with various numbers of cis-SNPs. In terms of the computation time (Table 1), all the four methods are very fast, but LMM is more efficient than other methods. The computation speeds of Lasso, ENET and BSLMM are comparable and can vary with the number of cross validation or the burn-in and MCMC sampling sizes.

**Table 1:** Computational time (in second) for the four models for predicting gene expression measurements

#SNP	PVE	Lasso	ENET	LMM	BSLMM
510	0.118	4.117 (0.203)	2.937 (0.073)	0.159 (0.148)	1.780 (1.789)
1375	0.002	6.594 (0.273)	5.345 (0.110)	0.560 (0.021)	3.895 (0.917)
2011	0.000	5.805 (0.172)	5.134 (0.100)	0.727 (0.076)	1.502 (0.841)
3045	0.357	8.623 (0.177)	7.992 (0.234)	1.097 (0.011)	8.286 (8.159)
4120	0.046	8.649 (0.282)	8.385 (0.227)	1.412 (0.073)	16.129 (8.792)
4953	0.523	10.019 (0.248)	9.772 (0.285)	1.621 (0.182)	7.626 (3.406)
5818	0.124	13.492 (0.199)	13.077 (0.237)	1.957 (0.057)	2.269 (0.854)

#SNP denotes the number of cis-SNPs included in this gene; PVE is the proportion of variance of gene expression explained by cis-SNPs; the tuning parameters of LASSO ENET are selected using 100-fold cross validation; BSLMM uses 10,000 Monte Carlo samplings after 2,000 burn-in samplings. The times are averaged across 20 replicates, and values in parentheses are the standard deviations

**Fig. 2:**
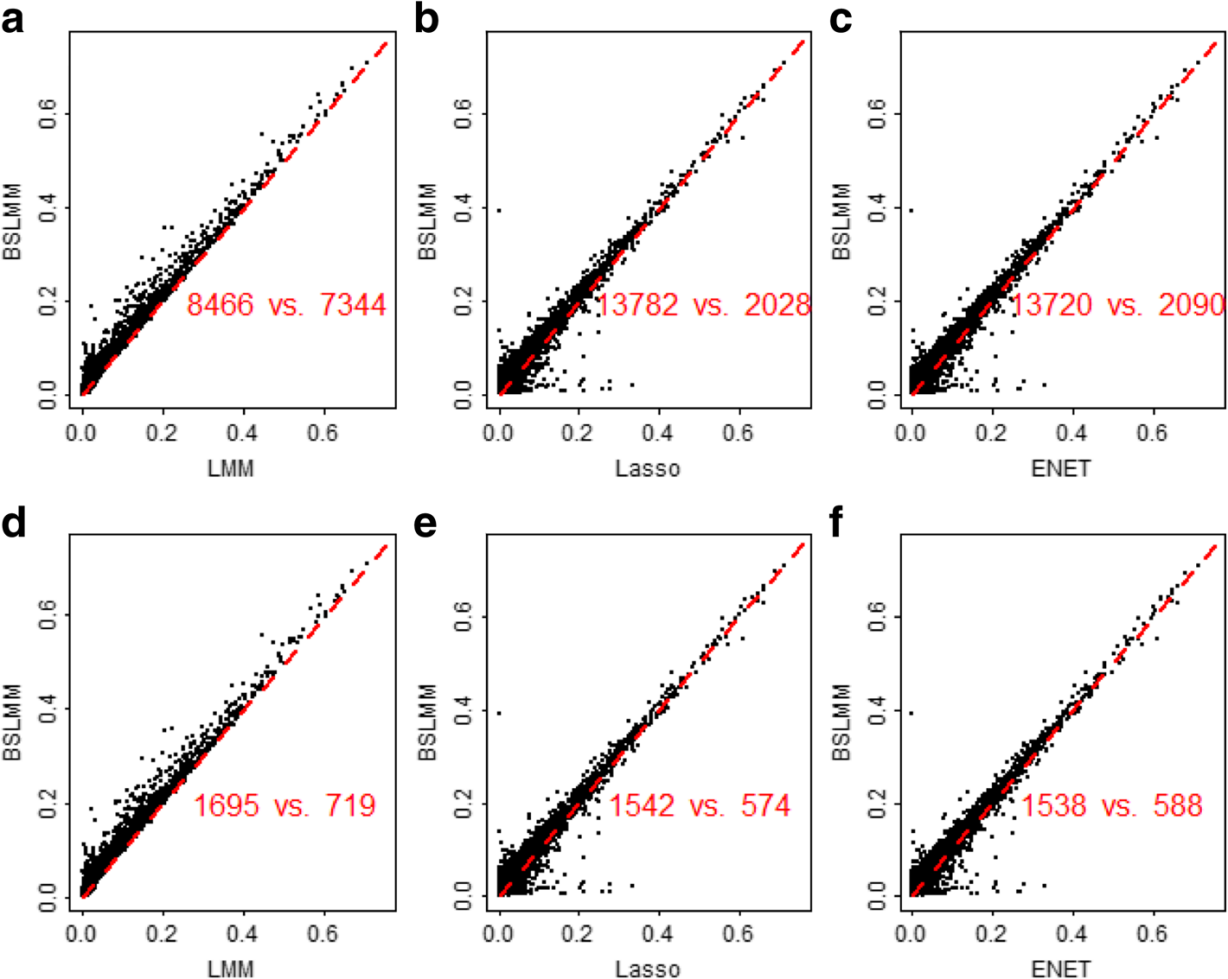
Comparison of the prediction performance of the four methods (i.e. Lasso, ENET, LMM and BSLMM) for the Geuvadis data. In each panel it lists the number of genes where BSLMM performs better and the number of genes where BSLMM performs worse; in the top (**a**)-(**c**), these numbers are computed across all the genes, and in the bottom (**d**)-(**f**) these numbers are computed across only the genes with *R* ^2^ ≥ 0.05

We now turn to the real application of the Geuvadis data. The predictive *R* ^2^ obtained from BSLMM versus other methods across all genes is presented in Fig. 2, where each panel also shows the number of genes for which BSLMM performs better and the number of genes for which BSLMM performs worse. In the top panel of Fig. 2a–c, these numbers are computed across all the genes, and in the bottom panel of Fig. 2d–f these numbers are computed across only the genes with predictive *R* ^2^ in the test data larger than 0.05. Table 2 lists the number of genes with a predictive *R* ^2^ above certain thresholds (from 0.05 to 0.60) for different methods. The four methods perform quite similarly to each other (Fig. 2 and Table 2). For example, the correlation coefficients of *R* ^2^ between BSLMM and other three methods are all above 0.970, and the correlation coefficient of *R* ^2^ between ENET and Lasso is even 0.999. Nevertheless, we can observe that BSLMM has a slightly higher predictive accuracy than other three methods. For instance, for these genes with *R* ^2^ ≥ 0.05 (Fig. 2d–f), the difference of *R* ^2^ between BSLMM and LMM, BSLMM and Lasso, and BSLMM and ENET has a mean of 8.49 × 10^−3^(standard deviation, or sd, =3.33 × 10^−4^), 7.67 × 10^−3^ (sd = 3.51 × 10^−4^), and 7.53 × 10^−3^ (sd = 3.46 × 10^−4^), respectively.

**Table 2:** Number of predictive genes passing the given *R* ^2^ threshold in the Geuvadis data and GenoExp data

threshold	Geuvadis data				GenoExp data			
	Lasso	ENET	LMM	BSLMM	Lasso	ENET	LMM	BSLMM
0.05	2252	2262	2447	2567	1785	1414	1560	1758
0.10	1144	1145	1145	1266	831	788	734	826
0.20	420	422	383	466	315	309	276	323
0.30	161	162	152	178	156	148	124	160
0.40	75	75	65	76	70	70	56	70
0.50	33	33	25	32	36	32	27	37
0.60	14	14	12	14	25	21	20	24

There are 15,810 and 15,427 genes in the Geuvadis data and GenoExp data, respectively. It can be seen that in both data sets when the given *R* ^2^ threshold is large (e.g. ≥0.30) the number of predictive genes passing that value in LMM is less than that of LASSO, ENET or BSLMM, implying that these highly predictive genes may have a sparse genetic architecture

More interestingly, it is observed from Fig. 2 and Table 2 that in the Geuvadis data there is little predictive difference among Lasso, ENET and BSLMM for highly predictive genes (e.g. with *R* ^2^ ≥ 0.30); whereas for these genes (*R* ^2^ ≥ 0.30) LMM achieves a smaller *R* ^2^. We further validate this finding using another gene expression data from GenoExp [52]. The GenoExp data was obtained from the HapMap Phase II data set [89], include 210 unrelated Epstein-Barr-virus-transformed lymphoblastoid cell line samples and 15,427 genes (with an average of about 304 cis-SNPs per gene). As before, for each gene the expression levels were quantile normalized to a standard normal distribution using the same procedure as in the Geuvadis data and were randomly divided into a training data with 80% individuals and a test data with the rest 20% individuals. We then fit Lasso, ENET, LMM and BSLMM on the training data and assessed their performance in the test data. For highly predictive genes (*R* ^2^ ≥ 0.30) in the GenoExp data, it can be also seen (Table 2) that LMM have a smaller *R* ^2^ compared with Lasso, ENET and BSLMM, which validates our previous finding and, together with the result of the Geuvadis data, supports the recent finding that these highly predictive genes may be influenced by a few of cis-SNPs with relatively large effect sizes [90]; in other words, these highly predictive genes may have sparse genetic architectures.

**Fig. 3:**
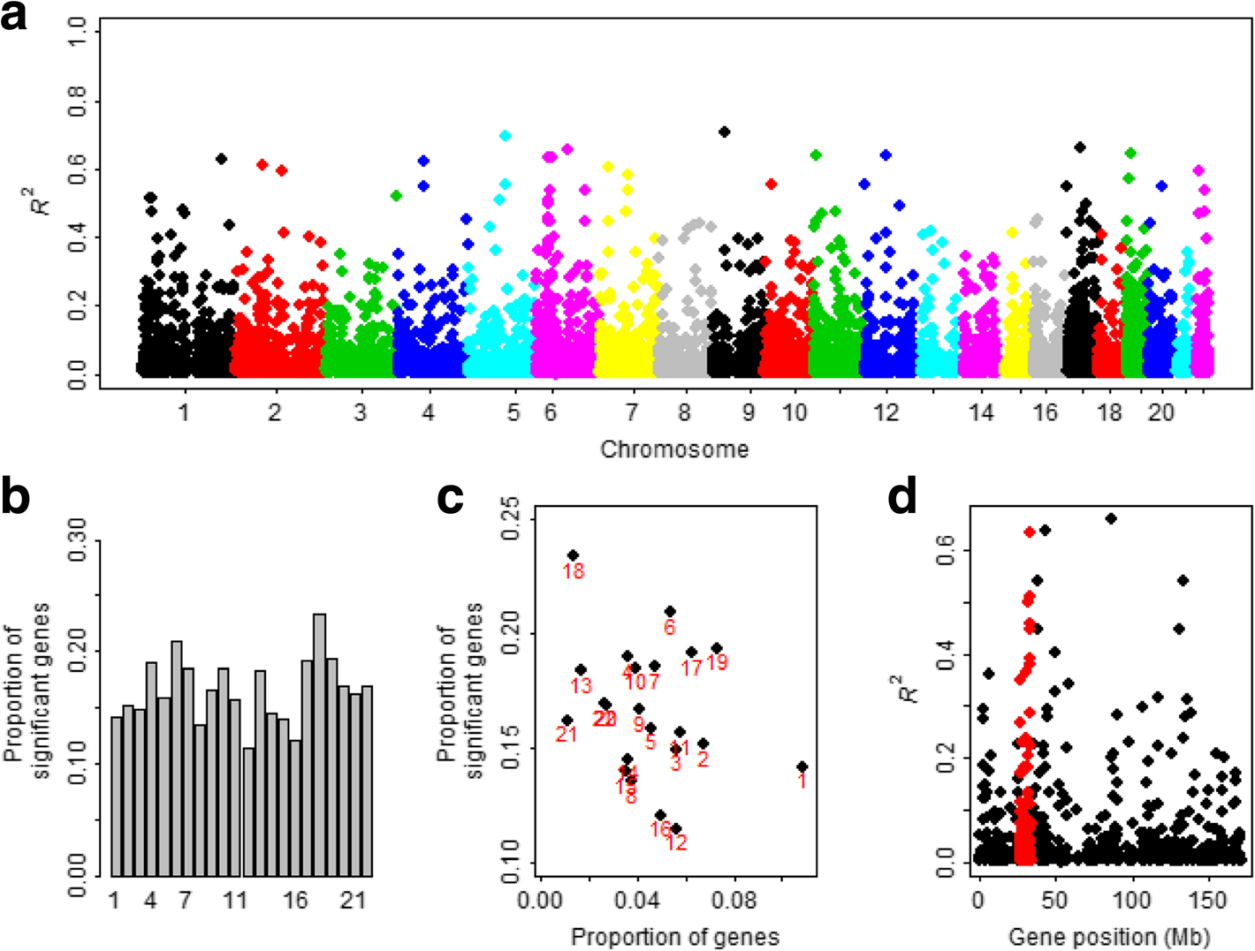
Distribution of *R* ^2^ of BSLMM for the Geuvadis data. **a** A Manhattan-type plot shows *R* ^2^ and gene positions across chromosomes, in which the y-axis is *R* ^2^ for each gene, the x-axis is the gene position and the various colors represent different chromosomes. **b** The barplot shows the proportion of predictive genes (*R* ^2^ ≥ 0.05) for each chromosome. **c** The scatter of the proportion of the predictive genes against the proportion of gene in each chromosome. **d** The *R* ^2^ pattern for the MHC region (chr6: 26-34 Mb); there are a total of 179 genes with *R* ^2^ ≥ 0.05 in chromosome 6, among which 45 are located on the MHC region (in red). The total length of chromosome 6 is about 171 Mb, and the length of the MHC region is 8 Mb. Then the enrichment-fold is 5.37, which is computed as the ratio of the proportion of predictive genes (i.e. 0.25 = 45/179) and the proportion of the length of MHC (i.e. 0.05 = 8/171), and is significantly higher (*P* = 1.79 × 10^−3^) than the average enrichment-fold (the median is 1.70) of other regions in chromosome 6

**Fig. 4:**
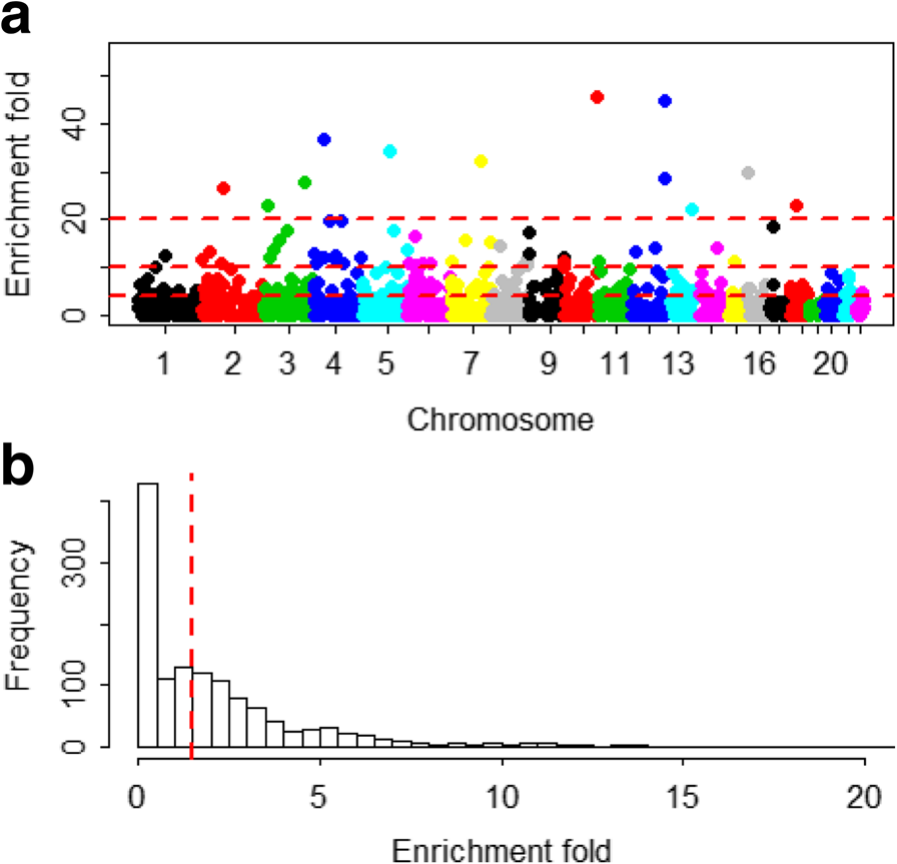
Enrichment-fold in 1,324 approximately independent LD blocks. **a** The enrichment-fold distributed across the chromosomes; the reference lines are 4, 10 and 20, respectively; (**b**) The histogram of enrichment-fold in 1,324 independent LD blocks; the median is 1.49 (indicating with red reference line) and the maximum is 299.82. The enrichment-fold is computed as the ratio of the proportion of predictive genes (i.e. *R* ^2^ ≥ 0.05) and the proportion of the length of that LD block

To further see whether the predictive genes show special pattern across the genome, we display four plots in Fig. 3. However, we do not observe any obvious clustering or enrichment of *R* ^2^ across the chromosomes (Fig. 3a and b), and we also do not see there is any clear relationship between the proportion of the predictive genes (*R* ^2^ ≥ 0.05) and the proportion of genes in each chromosome (Fig. 3c). The predictive genes are defined the genes with *R* ^2^ ≥ 0.05, which means that about 5% variation of gene expression is explained by only cis-SNPs and is selected arbitrarily to some extent; although other larger values can be also used and may lead to different results, the conclusions can not be changed. However, we indeed find enrichments of predictive genes in some special genetic regions. For example, for the MHC region of chromosome 6 (Fig. 3d), there are a total of 179 genes with *R* ^2^ ≥ 0.05 in chromosome 6, among which 45 are located in the MHC region. The total length of chromosome 6 is about 171 Mb, and the length of the MHC region is about 8 Mb (from 26 Mb to 34 Mb [91]). Then the enrichment-fold is 5.37, which is computed as the ratio of the proportion of predictive genes (i.e. 0.25 = 45/179) and the proportion of the length of MHC (i.e. 0.05 = 8/171), and is significantly higher than the average enrichment-fold (the median is 1.70) of other regions in chromosome 6 (*P* = 1.79 × 10^−3^ based on an approximate z test [92]). For the Geuvadis data we obtained 1,324 approximately independent blocks (1.6 Mb on average) (Fig. 4) of LD [51], with the median enrichment-fold being 1.49. Among these, there are 17 LD blocks with enrichment-fold ≥ 20 (Table 3), within which it has been identified by previous GWASs [93] that many SNPs are related to a lot of complex diseases and traits, including type 2 diabetes, aging-related traits, blood pressure, body mass index, bipolar disorder, Crohn’s disease, lung cancer, obesity, schizophrenia and coronary heart disease. Therefore, the enrichment of predictive genes in these LD blocks may provide important implications for the underlying functional basis of identified SNPs in GWASs.

**Table 3:** Enrichment-fold (≥20) of independent LD blocks in the Geuvadis data

Enrichment fold	#Identified SNPs	Chromosome	LD block	
			lower	upper
26.58	16	2	84,687,169	84,743,579
53.20	11	2	152,118,393	152,146,571
22.76	17	3	19,988,517	20,053,822
184.35	8	3	75,713,481	75,721,542
27.75	17	3	161,090,668	161,144,215
36.70	15	4	44,680,444	44,728,612
81.95	8	4	47,465,736	47,487,305
34.18	13	5	107,006,596	107,052,542
32.41	25	7	120,965,421	121,036,418
299.82	29	10	18,940,551	18,948,334
45.61	12	10	131,909,081	131,934,663
28.64	8	12	127,210,816	127,256,957
44.88	23	12	129,308,528	129,337,972
22.28	17	13	101,241,782	101,327,347
29.80	13	16	5,084,142	5,147,789
23.06	22	18	23,671,164	23,806,409
151.19	16	18	61,616,535	61,637,159

We obtained a total of 18,896 complete records (mainly including the information of disease/trait, chromosome id and position) of identified SNPs by GWASs from https://www.genome.gov/gwastudies/. We counted the number (given in the second column) of related SNPs within 1 Mb upstream and downstream regions near each LD block. These identified SNPs are extensively related to about 130 different types of complex diseases and traits. For example, in the first LD block (Chr2: 84,687,169-84,743,579), previous GWASs have discovered 16 associated SNPs, which, in terms of the catalog of published GWASs, are related to aging traits, protein quantitative trait loci, pulmonary function decline, IgG glycosylation, RR interval heart rate, the response to antipsychotic therapy, coronary artery calcification, prostate cancer, response to cytadine analogues cytosine arabinoside, bilirubin levels, orthostatic hypotension, breast cancer and conduct disorder

## Discussion and conclusions

In this paper we have explored to predict gene expression using only cis-SNPs and compared four prediction methods (i.e. Lasso, ENET, LMM and BSLMM). The four methods represent three types of prediction approaches that are widely used for genetic data in which the number for predictors (i.e. SNPs) is typically larger than the sample size [57, 62, 66, 94, 95]. Lasso and ENET assume the underlying model is sparse and only include important cis-SNPs into the model by regularization. In contrast to the sparsity, LMM assumes all cis-SNPs have impacts on the gene expression and thus is an explicit polygenic model. BSLMM combines the sparse model and LMM, and can have the benefits of both the models. Therefore, as shown in simulations the sparse model and LMM work well under individual model assumption, but become worse when their model assumptions are not met. On the other hand, BSLMM has a robust performance across different scenarios and is the best model or performs comparably with the best model.

Note that there are other risk prediction methods that are not considered here. For example, the Bayes-alphabet models [58, 59], which use slight different mixture priors from BSLMM and thus should have similar performance. BayesR [96] and Multi-BLUP [97] are more recently developed risk prediction methods, but they typically require more dense SNPs to achieve a better prediction accuracy, thus may improve little compared with BSLMM in the context of gene expression prediction. Besides single-trait prediction methods, multi-trait prediction approaches have also attracted significant recent attention. It has been shown that by leveraging shared genetic basis underlying correlated phenotypes multi-trait prediction approaches are typically more powerful than single-trait prediction methods [98–100]. Since multiple gene expression levels in an independent LD block may be highly correlated and have common genetic basis, analyzing a set of gene expression levels jointly using multi-trait approaches is expected to offer a potential to further increasing prediction accuracy. We will investigate this interesting problem in our further work.

In the application of the Geuvadis gene expression data, the four methods behave similarly; but it is very interesting that BSLMM and the two sparse models (i.e. Lasso and ENET) have a better performance for some genes that have high *R* ^2^ (e.g. ≥0.30), more importantly, this finding is further validated in an external data set, suggesting that these highly predictive genes may have sparse genetic architectures [90]. In the Geuvadis data, we also find that the predictive genes are enriched in some approximately independent LD blocks, meaning that for some special genome regions (e.g. MHC) in human [79] the gene expression values are more predictive relative to other regions, and thus can provide further useful insights for revealing the biological function of regulatory variants.

According to the computational efficiency, LMM is the fastest method; BSLMM, Lasso and ENET are computationally comparable. As we use the R package glmnet [75] to conduct Lasso and ENET, which may limit their utility for larger data set; but this limitation seems to not be a problem in the context of gene expression prediction using cis-SNPs, since currently the sample size of the gene expression data is relatively small. On the other hand, LMM and BSLMM are performed using the GEMMA software [58, 78], which can be applicable to large scale data set. Note that the computation time is dependent not only on implementational environment, computer language, the number of cis-SNPs and the sample sizes but also on other factors, for instance, the number of the cross-validation used in Lasso and ENET, and the burn-in steps and the posterior sampling steps in BSLMM.

Finally, we need to emphasize that like in [52] the prediction accuracies of these models are still low for most genes, although we discover some gene expression levels can be effectively predicted by cis-SNPs in the Geuvadis data. There may be other factors that are also responsible for gene expression, such as trans-SNPs and environmental factors. In summary, in this paper we have demonstrated that gene expression can be predicted with only cis-SNPs using well-developed prediction models that are commonly-used in GWASs and the prediction of gene expression can shed some light on the functional interpretation for these identified SNPs in GWASs.

## Abbreviations

BSLMM: Bayesian sparse linear mixed model
ENET: Elastic net
GWASs: Genome wide association studies
LD: Linkage disequilibrium
LMM: Linear mixed model
MCMC: Monte Carlo Markov chain
MHC: Major histocompatibility complex
PVE: Proportion of variance of explained
